# Recognizing the SINEs of Infection: Regulation of Retrotransposon Expression and Modulation of Host Cell Processes

**DOI:** 10.3390/v9120386

**Published:** 2017-12-18

**Authors:** William Dunker, Yang Zhao, Yu Song, John Karijolich

**Affiliations:** 1 Department of Pathology, Microbiology, and Immunology, Vanderbilt University School of Medicine, Nashville, TN 37232-2363, USA; william.dunker@vanderbilt.edu (W.D.); yang.zhao.1@vanderbilt.edu (Y.Z.); yu.song@vanderbilt.edu (Y.S.); 2 College of Pharmacy, Xinxiang Medical University, Xingxiang 453000, China; 3 Vanderbilt-Ingram Cancer Center, Nashville, TN 37232-2363, USA

**Keywords:** retrotransposons, SINEs, gene expression regulation, DNA virus infection, Murine Gammaherpesvirus 68 (MHV68), transcriptional interference, innate immunity

## Abstract

Short interspersed elements (SINEs) are a family of retrotransposons evolutionarily derived from cellular RNA polymerase III transcripts. Over evolutionary time, SINEs have expanded throughout the human genome and today comprise ~11% of total chromosomal DNA. While generally transcriptionally silent in healthy somatic cells, SINE expression increases during a variety of types of stresses, including DNA virus infection. The relevance of SINE expression to viral infection was largely unexplored, however, recent years have seen great progress towards defining the impact of SINE expression on viral replication and host gene expression. Here we review the origin and diversity of SINE elements and their transcriptional control, with an emphasis on how their expression impacts host cell biology during viral infection.

## 1. Introduction

Sequencing of the human genome has determined that approximately 1.5% is exonic, or protein coding [1,2]. While the remaining 98.5% was initially regarded as ‘junk’, it is now widely accepted that the noncoding portion of the genome plays a significant role in diverse cellular processes. The largest contributors to the remaining genome are transposable elements. Transposable elements are classified on the basis of their transposition mechanism. Class I elements, commonly referred to as retrotransposons, mobilize throughout the genome via transcription into RNA intermediates that are reverse-transcribed and inserted at new genomic locations. This mechanism of genomic expansion has been referred to as the “copy-and-paste” method [3]. In contrast, class II elements, or DNA transposons, do not use an RNA intermediate to amplify, but instead “cut-and-paste” themselves by endonucleolytic cleavage of the DNA at the elements’ terminal inverted repeats, and are inserted elsewhere in the genome.

Retrotransposons are by far the largest class of transposable elements in the human genome and currently comprise ~45% of the genomic sequence [4]. They can be classified into two major groups: those lacking long terminal repeats (LTRs), which include long and short interspersed elements (LINEs and SINEs, respectively), and those with LTRs, termed endogenous retroviruses (ERVs). Interestingly, ERVs are remnants of past retroviral infections established in the germline of ancestral species [5]. In contrast, SINE elements are derived from the retrotransposition of host encoded RNA polymerase (RNAP) III transcripts, such as tRNA and 7SL RNA [6,7,8,9]. The evolutionary biogenesis of LINE elements is much less clear. However, LINEs are the most transpositionally active elements in the human genome, and facilitate the mobilization of SINEs [10]. LINEs encode for two proteins, open reading frame (ORF) 1 and ORF2, that function as an RNA-binding protein, and an endonuclease and reverse transcriptase, respectively. Both activities are required for SINE mobilization [11,12,13]. 

SINEs are expressed in germ cells early during development; however, as the cells and the organism differentiate, the genes encoding SINEs become epigenetically silenced [14]. Interestingly, many groups, including our own, have demonstrated that SINEs regain transcriptional potential in various conditions of stress, including viral infection. The biological significance of their expression has only recently become apparent. Here, we review the origin and diversity of SINE elements and their transcriptional control, and discuss how DNA virus infection impacts their expression and thus influences host cell biology.

## 2. Structure and Diversity of SINEs

**Figure 1:**
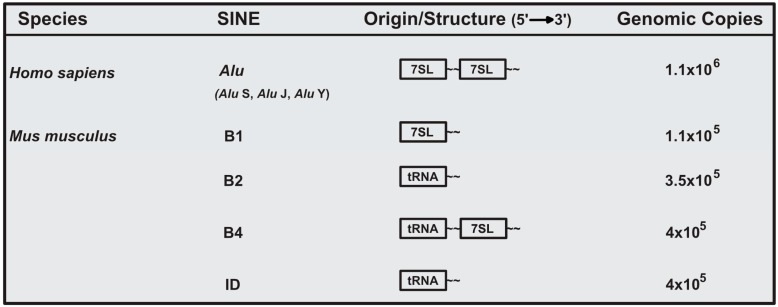
Origin and genomic abundance of major human and murine short interspersed element (SINE) families.

The murine and human genome are estimated to both contain ~1 million SINE loci, comprising ~11% of genomic sequence [1,2,15]. Given their ancient origin, SINE genomic sequence has been subjected to extensive mutational and evolutionary processes, and thus current SINE elements are highly diverse. In the *Mus musculus* genome, there are four major and unrelated SINE families. These families are B1, B2, B4, and identifier (ID) [16]. B1 elements are evolutionarily derived from 7SL RNA, the RNA component of the signal recognition particle. In contrast, B2 and ID SINEs are derived from ancestral tRNA species, while the B4 SINE is a chimeric tRNA-7SL derived SINE (Figure 1) [6,8,17]. Of the murine SINEs, B1 and B2 have been the most extensively investigated. 

In humans, the most abundant SINE is the *Alu* element, named for an *AluI* restriction site present within these repeats [18]. *Alu* elements are divided into three distinct subfamilies (*Alu* J, *Alu* S, and *Alu* Y). Evolutionarily, the J subfamily arose first, followed by the S, and Y subfamilies [10]. Similar to murine B1, *Alu* SINEs are evolutionarily derived from 7SL RNA (Figure 1). However, it is important to note that B1 and *Alu* evolved from independent retrotransposition events and are thus only related in terms of the RNA of origin [7]. 

**Figure 2:**
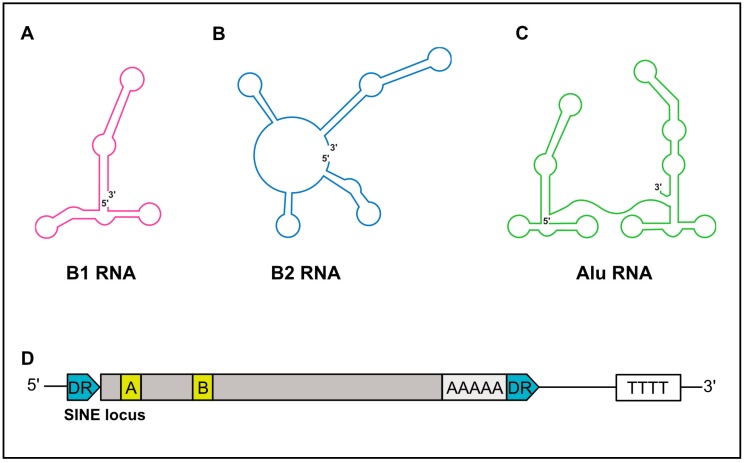
Schematic structures of SINE RNA and genomic loci. (**A**) Schematic secondary structures of B1 RNA, (**B**) B2 RNA, and (**C**) *Alu* RNA. 5′ and 3′ ends of RNA are denoted. (**D**) Depiction of a SINE genomic locus. SINEs are flanked by direct repeats (DRs, in blue). SINEs are driven by a type II RNA polymerase (RNAP) III promoter, within internal A and B boxes (yellow). A poly A sequence is often near the 3′-end. Transcription termination occurs at a downstream poly (T) sequence. Grey boxes represent the remaining SINE sequence.

SINEs are short, ranging in length from 75–500 nt, and possess a significant predicated secondary structure [19]. While B1 and B2 RNAs are monomeric in structure, *Alu* RNAs are dimeric and composed of two similar monomers separated by an internal A-rich sequence (Figure 2A–C) [20]. The 5′-end of SINE RNAs can contain a triphosphate moiety, in part explaining their ability to elicit cell-intrinsic immune responses (discussed below). However, some B2 SINE RNAs have been demonstrated to contain gamma-methylphosphate caps, while others are dephosphorylated to a monophosphate via dual specificity phosphatase 11 [21,22,23]. Importantly, these 5′-end features render human and murine SINEs void of a cap structure capable of engaging the translation machinery via canonical mechanisms, and are thus noncoding.

Mobilization of SINEs is facilitated by LINEs, which recognize polyA sequences within human and murine SINEs. Whereas *Alu* elements encode an A-rich sequence near their 3′-end, B2 SINEs can acquire their polyA tail post-transcriptionally [24,25]. The post-transcriptional polyadenylation of B2 SINEs is facilitated by canonical mRNA polyadenylation sequences, such as a polyadenylation signal (PAS; 5′-AAUAAA-3′) near their 3′-end [25].

## 3. Transcriptional Regulation of SINEs

SINE elements are transcribed by RNAP III. RNAP III is a multi-subunit polymerase primarily responsible for the synthesis of essential housekeeping small noncoding RNAs, including tRNAs, 5S rRNA, and U6 snRNA [26]. RNAP III genes are divided into three types (I–III), distinguishable by the sequence motifs that facilitate recruitment of the RNAP III transcription complex [27]. SINEs, similar to tRNA, have an internal type II promoter, comprised of internal promoter elements termed the A box and B box (Figure 1D) [28,29,30]. For transcription of SINEs to occur, the A and B boxes initially recruit the multisubunit transcription factor complex TFIIIC [31]. TFIIIC then recruits the TFIIIB complex, which is composed of three polypeptides, one of which is the TATA-box-binding protein (TBP), to the promoter [32,33]. The binding of TFIIIB to the promoter in turn allows the recruitment of RNAP III, mainly through protein–protein interactions with TFIIIB, although contacts with TFIIIC may also contribute. Termination of RNAP III-transcribed SINEs occurs in poly-T sequences in close proximity to their 3′ end [34,35,36,37,38]. 

SINE expression is regulated epigenetically. A common epigenetic mark associated with transcriptional repression is CpG methylation [39]. Early investigations suggested SINE expression was regulated by CpG methylation. For instance, treatment of HeLa cells with the DNA methylation inhibitor 5-azacytidine robustly increased SINE expression [40]. Additionally, methylation of CpG motifs in the A box of SINE promoters prevented in vitro transcription [41]. These experiments align well with the observation that nearly 25% of the ~30 million CpG sites in the human genome are within *Alu* sequences, and the majority of these motifs are methylated [41,42]. However, recent investigations into the mechanism of SINE repression have revealed no role for CpG methylation. For instance, the presence of CpG methylation and methyl-CpG-binding proteins, such as Methyl-CpG Binding Protein 2 (MeCP2), Methyl-CpG Binding Domain Protein 1 (MBD1), and MBD2, do not preclude the binding of RNAP III to *Alu* loci [43]. Additionally, 5-azacytidine treatment does not increase RNAP III binding to SINEs. In contrast, *Alu* expression was found to be regulated by histone methylation, specifically H3K9me3 [43]. The SUV39 family member, SUV39H1, was found to mediate H3K9me3 at bound loci, and treatment of HeLa cells with chaetocin, a selective inhibitor of the SUV39 family, increased RNAP III occupancy at *Alu* loci. A role of H3K9me3 in regulating murine SINE expression is further suggested by data demonstrating that a dominant-negative SUV39H mutant stimulates the expression of B1 and B2 RNA in murine cells [44].

## 4. Induction and Consequences of SINE Expression

In embryonic stem cells, murine germ cells, and early during murine embryonic development, SINE elements are transcriptionally active [45,46,47,48]. In contrast, in healthy somatic cells, SINE elements are typically transcriptionally repressed. However, SINE RNA expression can be induced when cells are subjected to chemical and biological stressors.

SINE RNAs are localized within both the cytoplasm and nucleus and can influence various aspects of cell biology depending on their localization. For example, in the cytoplasm, *Alu* RNA can form stable complexes with protein kinase R (PKR), and depending on concentration either activate or repress PKR activity [49,50]. In contrast, nuclear *Alu* and B2 RNA can repress gene expression by interacting directly with RNAP II, preventing it from establishing contacts with the promoter during closed complex formation [51,52,53]. Enhancer Of Zeste Homolog 2 (EZH2), a polycomb protein traditionally associated with histone methyltransferase activity and the repression of gene expression, binds B2 RNAs during the heat shock response and initiates their endonucleolytic cleavage [54]. EZH2-mediated B2 destruction alleviates the block in gene expression during the heat shock response.

SINE expression is also induced during a variety of DNA viral infections. For instance, adenovirus infection increases the expression of *Alu* elements [55]. Viral early proteins, including E1a, E1b, ORF3, and ORF6 have been suggested to mediate *Alu* induction. During parvovirus minute virus of mice (MVM) infection, B1 and B2 SINEs are upregulated continuously over the course of the infection [56]. The major non-structural (NS) protein of MVM, NS1, induces the expression of the elements through increased RNAP III activity. Additionally, transformation of murine cells with Simian virus 40 (SV40) enhances B2 expression [57]. Interestingly, the levels of 5S rRNA, another RNAP III transcribed RNA, were not increased, suggesting specificity in the transcriptional response. It is tempting to speculate that SINE expression may be involved in viral transformation of cells. Infection of HeLa cells with herpes simplex virus-1 (HSV-1) has also been shown to enhance *Alu* transcription, and the viral protein ICP27 (infected cell protein 27) has been suggested to mediate this effect [58,59]. Unfortunately, a biological role for SINE RNA expression during DNA virus infection was not elucidated in any of the above-mentioned studies.

**Figure 3:**
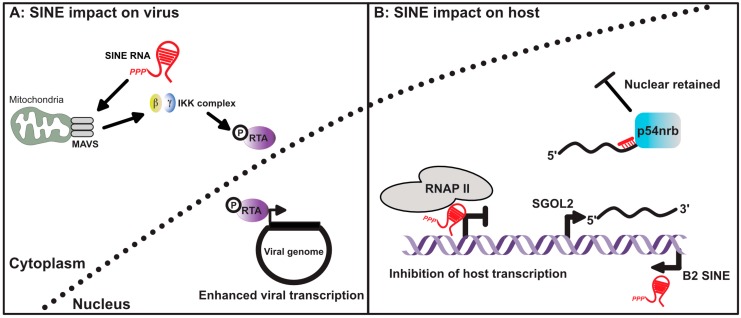
SINE RNA influences gene expression in virus infected cells. (**A**) Cytoplasmic SINE RNAs can activate the mitochondrial antiviral-signaling protein (MAVS), subsequently activating the IKK complex and promoting phosphorylation of the viral transcription factor RTA. Phosphorylation of RTA enhances its function as a viral transcription factor. (**B**) Nuclear localized SINE RNA can also inhibit host gene transcription by preventing RNAP II from properly engaging promoters. Transcriptional activation of SINEs within the 3’ untranscribed regions (UTRs) of host mRNAs can also lead to nuclear retention of the antisense mRNA. This occurs as a result of intermolecular RNA–RNA interactions between the mRNA and expressed SINE RNA, leading to the recruitment of p54nrb.

Our understanding of how viral-mediated SINE expression impacts host cell biology has only recently become apparent, and is best understood in the context of murine gamma-herpesvirus-68 (MHV68) infection (Figure 2). MHV68 rapidly induces B1 and B2 SINEs following in vitro and in vivo infection [60]. SINE expression during MHV68 infection is likely mediated by several viral proteins as a plasmid-based overexpression screen did not identify a single viral gene product sufficient to induce SINE expression. Expressed SINEs rapidly activate nuclear factor (NF)-κB in a manner partially dependent on the presence of the mitochondrial antiviral-signaling protein (MAVS), which functions as an adapter for the RIG-I-like receptors [61]. Unexpectedly, depletion of B2 SINEs from infected cells resulted in a viral replication defect, suggesting MHV68 co-opted the B2-mediated NF-kB response. Indeed, further analyses revealed that MHV68 hijacks the inhibitor of nuclear NF-κB kinase subunit β (IKKβ) kinase within the NF-κB pathway, and redirects it to phosphorylate and enhance the activity of the major viral transcriptional regulator replication and transcription activator (RTA), thus enhancing viral gene expression and replication (Figure 3) [60,62]. The ability of SINEs to activate NF-κB during infection is consistent with recent observations made in patients with age-related macular degeneration and systemic lupus erythematosus where aberrant expression of *Alu* RNAs promotes the activation of NF-κB and an inflammasome, as well as cytokine release [63,64,65].

A central question regarding SINE expression is which of the ~1 million SINE loci are transcriptionally active. Historically, identifying transcriptionally active SINE loci relied on either chromatin immunoprecipitation sequencing (ChIP-seq) analyses of RNAP III or the use of extensive computational algorithms to analyze RNA-seq data. However, these techniques have significant limitations, including the extremely high copy number and sequence similarity among SINE elements of the same family, and the frequent location of SINE elements within introns or untranslated regions of RNAP II transcripts. To address these issues, Karijolich et al. recently developed a novel sequencing method, called SINE-seq, capable of directly identifying transcriptionally active SINE loci [66]. Profiling B2 SINE expression during MHV68 infection revealed 28,270 transcriptionally active B2 loci. Surprisingly, ~50% of the expressed B2 RNAs identified were transcribed from within or near annotated RNAP II genes, including in the antisense orientation within 3′ untranscribed regions (UTRs) of mRNAs, raising the possibility that they can impact the post-transcriptional fate of the overlapping RNA. Indeed, a B2 RNA expressed from an antisense element within the Shugoshin 2 (SGOL2) 3’UTR was shown to base pair with SGOL2 mRNA, leading to nuclear retention of SGOL2 through a mechanism involving the protein p54nrb (Figure 2). This represents a novel pathway for the selective regulation of mRNA export during stress via retrotransposon activation.

It is likely that the transcriptional activation of SINE loci within RNAP II genes has additional consequences. For example, the first intron of the *Polr3e* gene has an antisense mammalian interspersed repeat (MIR) element that is occupied by RNAP III [67]. Interestingly, a minor, but significant accumulation of RNAP II can be observed immediately upstream of the intronic RNAP III, suggesting transcriptional interference between the opposing polymerases. Indeed, when the MIR was removed using CRISPR/Cas9, *Polr3e* expression increased, and the accumulation of intronic RNAP II and III were no longer observed. Although RNAP II and III collisions have not been studied, transcriptional interference between convergent RNAP II molecules has [68]. RNAP II molecules that collide are unable to bypass one another and do not disassociate from the genome. In order for transcription to resume, the polymerases must be cleared. This is accomplished by ubiquitination of one of the polymerase molecules, leading to its degradation via the proteasome [69,70,71,72]. Though the ubiquitin-dependent clearance is slow, it removes the block and allows for transcription to resume. How RNAP II–III collisions are resolved is unknown, though it is reasonable to hypothesize that it is by a similar mechanism. To date, RNAP II–III collisions have not been observed during viral infection. However, given that SINE-seq has defined a comprehensive set of active B2 loci, MHV68 infection may represent an ideal opportunity to identify RNAP II–III collisions and characterize the mechanism by which they are resolved. 

## 5. Closing Remarks

SINE RNAs are transcriptionally activated during several viral infections and we are just beginning to define ways by which their expression impacts host cell biology. To date, SINE expression has only been examined during DNA viral infections; however, it is likely that other infections, including prokaryotic and eukaryotic pathogens, as well as RNA viruses, induce SINE expression. Whether SINE RNA impacts these host pathogen interactions is an important area of investigation. It will also be interesting to determine whether the same SINE loci are induced in response to distinct pathogen challenges. 

SINEs are still active transpositionally, and whether they are mobilized during infection is unclear. However, a recent analysis demonstrated an increase in *Alu* DNA copy number upon HIV-1-infection of primary CD4+ T cells [73]. Given that SINE transposition is inherently a mutagenic event, it will be important for future studies to determine if SINE mobilization is a common property of infection. Viruses have provided great insight into many aspects of mammalian biology. We anticipate this will continue and that viral infections will lead the way in defining novel aspects of SINE biology and their impact on host function.
